# (1-Naphthyl­meth­yl)ammonium chloride

**DOI:** 10.1107/S160053681000334X

**Published:** 2010-02-03

**Authors:** Ali R. Salimi, Mahmood Azizpoor Fard, Hossein Eshtiagh-Hosseini, Mostafa M. Amini, Hamid R. Khavasi

**Affiliations:** a Department of Chemistry, Ferdowsi University of Mashhad, Mashhad 917791436, Iran; b Department of Chemistry, General Campus, Shahid Beheshti University, Tehran 1983963113, Iran

## Abstract

The reaction of 1-naphthyl­methyl­amine and hydro­chloric acid in a 1:1 molar ratio resulted in the formation of the 1:1 proton-transfer compound, C_11_H_12_N^+^·Cl^−^. In the crystal, the ions are linked by N—H⋯Cl hydrogen bonds into a sheet pattern in the *ab* plane such that each Cl^−^ ion is bonded to three NH groups from the naphthylmethylammonium ion.

## Related literature

For 1-naphthyl­methyl­ammonium salts, see: Sada *et al.* (2004 ▶).
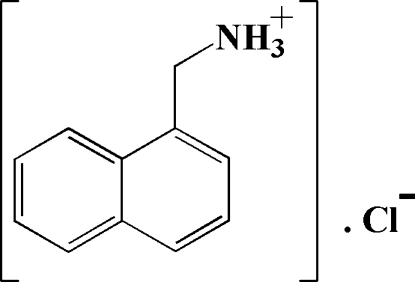


## Experimental

### 

#### Crystal data

C_11_H_12_N^+^·Cl^−^
*M* *_r_* = 193.67
Monoclinic, 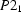

*a* = 5.3395 (7) Å
*b* = 9.3355 (15) Å
*c* = 10.1432 (13) Å
β = 100.864 (10)°
*V* = 496.55 (12) Å^3^
*Z* = 2
Mo *K*α radiation
μ = 0.34 mm^−1^
*T* = 298 K
0.35 × 0.13 × 0.11 mm

#### Data collection

Stoe IPDS II diffractometer
Absorption correction: numerical (*X-RED* and *X-SHAPE*; Stoe & Cie, 2005 ▶) *T*_min_ = 0.952, *T*_max_ = 0.968
5801 measured reflections
2677 independent reflections
2098 reflections with *I* > 2σ(*I*)
*R*_int_ = 0.065

#### Refinement

*R*[*F* ^2^ > 2σ(*F* ^2^)] = 0.054
*wR*(*F* ^2^) = 0.100
*S* = 1.19
2677 reflections
130 parameters
1 restraint
H atoms treated by a mixture of independent and constrained refinement
Δρ_max_ = 0.28 e Å^−3^
Δρ_min_ = −0.17 e Å^−3^
Absolute structure: Flack (1983 ▶), 245 Friedel pairs
Flack parameter: 0.09 (10)

### 

Data collection: *X-AREA*; cell refinement: *X-AREA*; data reduction: *X-AREA*; program(s) used to solve structure: *SHELXS97* (Sheldrick, 2008 ▶); program(s) used to refine structure: *SHELXL97* (Sheldrick, 2008 ▶); molecular graphics: *ORTEP-3 for Windows* (Farrugia, 1997 ▶); software used to prepare material for publication: *WinGX* (Farrugia, 1999 ▶).

## Supplementary Material

Crystal structure: contains datablocks global, I. DOI: 10.1107/S160053681000334X/om2314sup1.cif

Structure factors: contains datablocks I. DOI: 10.1107/S160053681000334X/om2314Isup2.hkl

Additional supplementary materials:  crystallographic information; 3D view; checkCIF report

## Figures and Tables

**Table 1:** Hydrogen-bond geometry (Å, °)

*D*—H⋯*A*	*D*—H	H⋯*A*	*D*⋯*A*	*D*—H⋯*A*
N1—H1*C*⋯Cl1	0.86 (5)	2.37 (5)	3.226 (3)	172 (3)
N1—H1*D*⋯Cl1^i^	0.94 (4)	2.27 (4)	3.187 (3)	164 (3)
N1—H1*E*⋯Cl1^ii^	0.92 (4)	2.29 (4)	3.172 (3)	161 (3)

Symmetry codes: (i) 
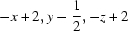
; (ii) 
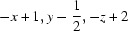
.

**Fig. 1.**
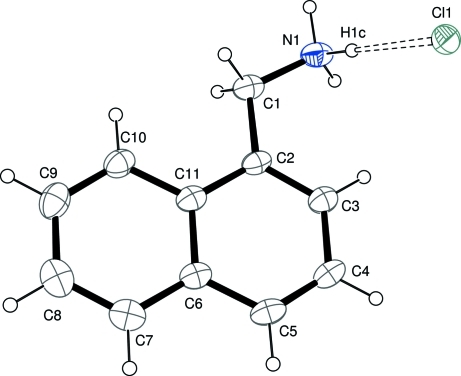
The molecular staucture with the atom-numbering scheme. Displacement ellipsoids are drawn at 30% probability level.

**Fig. 2.**
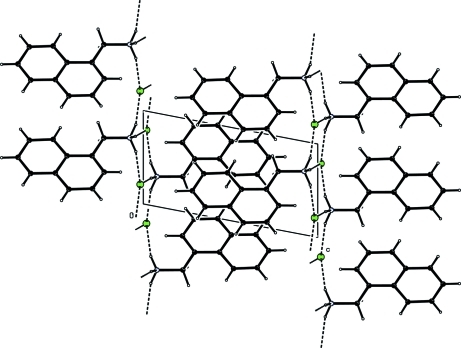
A packing diagram of the title compound in thr b-direction. Hydrogen bonds are shown as dashed lines.

## supplementary crystallographic information

### Comment

1-naphthylmethylamine has been recognized as a suitable agent in the synthesis of proton-transfer systems (Sada *et al.*,  2004). We report here the synthesis and characterization of the title salt, 1-naphthylmethylammonium chloride. The structure shows the presence of a 1-naphthylmethylammonium species that arises from the protonation of the amine group (Fig. 1). Hydrogen bonds play a very important role in the structure. As it is clear from Figure 2, chloride atoms engage in three hydrogen bonds with the amine group in which the chloride is in the center of triangle from three H atoms from three different cations.

### Experimental

A solution of 2.5 ml of 2M hydrochloric acid was added to a solution of 5 mmol 1-naphthalenemethylamine (0.73 ml) in 30 ml pyridine. The resulting solution was stirred at 373 K for 5 h and at ambient temperature for 24 h. A pale brown solution resulted. After drying the remaining brown solid was dissolved in pure methanol. X-ray quality crystals were obtained by slow evaporation at room temperature.

### Refinement

All of the H atoms bonded to C were positioned geometrically with C—H = 0.93 and 0.97Å for aromatic ring and CH_2_ hydrogen atoms respectively, and constrained to ride on their parent atoms, with U_iso_(H) = 1.2U_eq_(C). NH_3_ hydrogens atoms were positioned from Fourier map and freely refined.

### Crystal data

**Table:**

C_11_H_12_N^+^·Cl^−^	*F*(000) = 204
*M**_r_* = 193.67	*D*_x_ = 1.295 Mg m^−^^3^
Monoclinic, *P*2_1_	Mo *K*α radiation, λ = 0.71073 Å
Hall symbol: P 2yb	Cell parameters from 1056 reflections
*a* = 5.3395 (7) Å	θ = 2.0–29.2°
*b* = 9.3355 (15) Å	µ = 0.34 mm^−^^1^
*c* = 10.1432 (13) Å	*T* = 298 K
β = 100.864 (10)°	Prism, colorless
*V* = 496.55 (12) Å^3^	0.35 × 0.13 × 0.11 mm
*Z* = 2	

### Data collection

**Table:**

Stoe IPDS II diffractometer	2098 reflections with *I* > 2σ(*I*)
rotation method scans	*R*_int_ = 0.065
Absorption correction: numerical (*X-RED* and *X-SHAPE*; Stoe & Cie, 2005)	θ_max_ = 29.2°, θ_min_ = 2.0°
*T*_min_ = 0.952, *T*_max_ = 0.968	*h* = −7→6
5801 measured reflections	*k* = −12→12
2677 independent reflections	*l* = −13→13

### Refinement

**Table:**

Refinement on *F*^2^	H atoms treated by a mixture of independent and constrained refinement
Least-squares matrix: full	*w* = 1/[σ^2^(*F*_o_^2^) + (0.0155*P*)^2^ + 0.2107*P*] where *P* = (*F*_o_^2^ + 2*F*_c_^2^)/3
*R*[*F*^2^ > 2σ(*F*^2^)] = 0.054	(Δ/σ)_max_ = 0.002
*wR*(*F*^2^) = 0.100	Δρ_max_ = 0.28 e Å^−^^3^
*S* = 1.19	Δρ_min_ = −0.17 e Å^−^^3^
2677 reflections	Absolute structure: Flack (1983), 1245 Friedel pairs
130 parameters	Flack parameter: 0.09 (10)
1 restraint	

### Special details

**Table:**

Geometry. All esds (except the esd in the dihedral angle between two l.s. planes) are estimated using the full covariance matrix. The cell esds are taken into account individually in the estimation of esds in distances, angles and torsion angles; correlations between esds in cell parameters are only used when they are defined by crystal symmetry. An approximate (isotropic) treatment of cell esds is used for estimating esds involving l.s. planes.

### Fractional atomic coordinates and isotropic or equivalent isotropic displacement parameters (Å^2^)

**Table:**

	*x*	*y*	*z*	*U*_iso_*/*U*_eq_	
C1	0.6260 (6)	0.3230 (3)	0.7737 (3)	0.0472 (8)	
H1A	0.5852	0.2242	0.7487	0.057*	
H1B	0.7793	0.3481	0.7406	0.057*	
C2	0.4110 (5)	0.4171 (3)	0.7057 (3)	0.0387 (6)	
C3	0.2613 (6)	0.4950 (3)	0.7758 (3)	0.0442 (7)	
H3	0.2941	0.4919	0.8691	0.053*	
C4	0.0597 (6)	0.5793 (4)	0.7084 (4)	0.0505 (8)	
H4	−0.0375	0.6326	0.7577	0.061*	
C5	0.0047 (6)	0.5840 (3)	0.5736 (3)	0.0501 (8)	
H5	−0.1313	0.6396	0.531	0.06*	
C6	0.1509 (5)	0.5057 (3)	0.4954 (3)	0.0409 (7)	
C7	0.0961 (7)	0.5081 (3)	0.3544 (4)	0.0529 (9)	
H7	−0.0423	0.561	0.3104	0.063*	
C8	0.2421 (8)	0.4342 (4)	0.2810 (4)	0.0591 (9)	
H8	0.2043	0.4373	0.1877	0.071*	
C9	0.4484 (7)	0.3540 (4)	0.3465 (4)	0.0571 (9)	
H9	0.5481	0.3039	0.2962	0.069*	
C10	0.5064 (6)	0.3476 (3)	0.4826 (4)	0.0486 (8)	
H10	0.6454	0.2933	0.5238	0.058*	
C11	0.3593 (5)	0.4221 (3)	0.5631 (3)	0.0395 (6)	
N1	0.6799 (6)	0.3336 (3)	0.9219 (3)	0.0495 (7)	
H1C	0.708 (8)	0.418 (5)	0.955 (4)	0.069 (12)*	
H1D	0.835 (7)	0.288 (3)	0.957 (3)	0.046 (9)*	
H1E	0.546 (8)	0.298 (4)	0.957 (4)	0.065 (12)*	
Cl1	0.79683 (14)	0.66025 (10)	1.01767 (8)	0.04759 (18)	

### Atomic displacement parameters (Å^2^)

**Table:**

	*U*^11^	*U*^22^	*U*^33^	*U*^12^	*U*^13^	*U*^23^
C1	0.0375 (17)	0.0408 (16)	0.062 (2)	0.0020 (14)	0.0058 (15)	0.0015 (15)
C2	0.0302 (14)	0.0284 (13)	0.0570 (18)	0.0001 (11)	0.0074 (13)	0.0007 (12)
C3	0.0404 (16)	0.0394 (15)	0.0531 (19)	0.0020 (13)	0.0096 (14)	0.0017 (13)
C4	0.0441 (18)	0.0429 (16)	0.067 (2)	0.0118 (14)	0.0171 (16)	0.0025 (16)
C5	0.0401 (17)	0.0416 (16)	0.068 (2)	0.0088 (14)	0.0095 (16)	0.0115 (15)
C6	0.0335 (16)	0.0328 (13)	0.056 (2)	−0.0042 (11)	0.0083 (13)	0.0032 (13)
C7	0.050 (2)	0.0468 (18)	0.060 (2)	−0.0081 (15)	0.0048 (16)	0.0078 (16)
C8	0.069 (2)	0.056 (2)	0.053 (2)	−0.0188 (19)	0.0126 (18)	−0.0009 (17)
C9	0.061 (2)	0.0467 (18)	0.069 (2)	−0.0062 (16)	0.0242 (19)	−0.0125 (17)
C10	0.0424 (18)	0.0398 (16)	0.064 (2)	0.0014 (13)	0.0104 (16)	−0.0065 (15)
C11	0.0308 (14)	0.0318 (13)	0.0561 (18)	−0.0066 (11)	0.0087 (13)	−0.0008 (13)
N1	0.0387 (16)	0.0421 (16)	0.0640 (19)	0.0032 (13)	0.0002 (14)	0.0029 (14)
Cl1	0.0426 (3)	0.0466 (3)	0.0527 (4)	−0.0046 (4)	0.0067 (3)	−0.0113 (4)

### Geometric parameters (Å, °)

**Table:**

C1—N1	1.480 (5)	C6—C11	1.425 (4)
C1—C2	1.506 (4)	C7—C8	1.363 (5)
C1—H1A	0.97	C7—H7	0.93
C1—H1B	0.97	C8—C9	1.393 (5)
C2—C3	1.374 (4)	C8—H8	0.93
C2—C11	1.421 (4)	C9—C10	1.357 (5)
C3—C4	1.402 (4)	C9—H9	0.93
C3—H3	0.93	C10—C11	1.418 (4)
C4—C5	1.344 (5)	C10—H10	0.93
C4—H4	0.93	N1—H1C	0.86 (4)
C5—C6	1.416 (4)	N1—H1D	0.94 (3)
C5—H5	0.93	N1—H1E	0.92 (4)
C6—C7	1.405 (5)		
			
N1—C1—C2	114.3 (3)	C8—C7—C6	121.1 (3)
N1—C1—H1A	108.7	C8—C7—H7	119.4
C2—C1—H1A	108.7	C6—C7—H7	119.4
N1—C1—H1B	108.7	C7—C8—C9	119.6 (3)
C2—C1—H1B	108.7	C7—C8—H8	120.2
H1A—C1—H1B	107.6	C9—C8—H8	120.2
C3—C2—C11	119.2 (3)	C10—C9—C8	121.2 (3)
C3—C2—C1	122.7 (3)	C10—C9—H9	119.4
C11—C2—C1	118.1 (3)	C8—C9—H9	119.4
C2—C3—C4	120.9 (3)	C9—C10—C11	121.3 (3)
C2—C3—H3	119.6	C9—C10—H10	119.4
C4—C3—H3	119.6	C11—C10—H10	119.4
C5—C4—C3	121.0 (3)	C10—C11—C2	123.2 (3)
C5—C4—H4	119.5	C10—C11—C6	117.3 (3)
C3—C4—H4	119.5	C2—C11—C6	119.5 (3)
C4—C5—C6	121.0 (3)	C1—N1—H1C	116 (3)
C4—C5—H5	119.5	C1—N1—H1D	110.2 (19)
C6—C5—H5	119.5	H1C—N1—H1D	101 (3)
C7—C6—C5	122.0 (3)	C1—N1—H1E	111 (2)
C7—C6—C11	119.5 (3)	H1C—N1—H1E	106 (4)
C5—C6—C11	118.4 (3)	H1D—N1—H1E	113 (3)
			
N1—C1—C2—C3	−6.2 (4)	C8—C9—C10—C11	0.1 (5)
N1—C1—C2—C11	175.2 (3)	C9—C10—C11—C2	179.2 (3)
C11—C2—C3—C4	−0.1 (5)	C9—C10—C11—C6	−1.0 (5)
C1—C2—C3—C4	−178.8 (3)	C3—C2—C11—C10	178.7 (3)
C2—C3—C4—C5	1.2 (5)	C1—C2—C11—C10	−2.6 (4)
C3—C4—C5—C6	−0.9 (5)	C3—C2—C11—C6	−1.1 (4)
C4—C5—C6—C7	179.5 (3)	C1—C2—C11—C6	177.6 (3)
C4—C5—C6—C11	−0.4 (5)	C7—C6—C11—C10	1.6 (4)
C5—C6—C7—C8	178.7 (3)	C5—C6—C11—C10	−178.5 (3)
C11—C6—C7—C8	−1.4 (4)	C7—C6—C11—C2	−178.5 (3)
C6—C7—C8—C9	0.4 (5)	C5—C6—C11—C2	1.4 (4)
C7—C8—C9—C10	0.2 (5)		

### Hydrogen-bond geometry (Å, °)

**Table:**

*D*—H···*A*	*D*—H	H···*A*	*D*···*A*	*D*—H···*A*
N1—H1C···Cl1	0.86 (5)	2.37 (5)	3.226 (3)	172 (3)
N1—H1D···Cl1^i^	0.94 (4)	2.27 (4)	3.187 (3)	164 (3)
N1—H1E···Cl1^ii^	0.92 (4)	2.29 (4)	3.172 (3)	161 (3)

Symmetry codes: (i) −*x*+2, *y*−1/2, −*z*+2; (ii) −*x*+1, *y*−1/2, −*z*+2.
